# Reversible Platelet Integrin αIIbβ3 Activation and Thrombus Instability

**DOI:** 10.3390/ijms232012512

**Published:** 2022-10-19

**Authors:** Jinmi Zou, Frauke Swieringa, Bas de Laat, Philip G. de Groot, Mark Roest, Johan W. M. Heemskerk

**Affiliations:** Synapse Research Institute Maastricht, Koningin Emmaplein 7, 6217 KD Maastricht, The Netherlands

**Keywords:** ADP, collagen, fibrinogen, integrin, platelets, thrombin

## Abstract

Integrin αIIbβ3 activation is essential for platelet aggregation and, accordingly, for hemostasis and arterial thrombosis. The αIIbβ3 integrin is highly expressed on platelets and requires an activation step for binding to fibrinogen, fibrin or von Willebrand factor (VWF). A current model assumes that the process of integrin activation relies on actomyosin force-dependent molecular changes from a bent-closed and extended-closed to an extended-open conformation. In this paper we review the pathways that point to a functional reversibility of platelet αIIbβ3 activation and transient aggregation. Furthermore, we refer to mouse models indicating that genetic defects that lead to reversible platelet aggregation can also cause instable thrombus formation. We discuss the platelet agonists and signaling pathways that lead to a transient binding of ligands to integrin αIIbβ3. Our analysis points to the (autocrine) ADP P2Y_1_ and P2Y_12_ receptor signaling via phosphoinositide 3-kinases and Akt as principal pathways linked to reversible integrin activation. Downstream signaling events by protein kinase C, CalDAG-GEFI and Rap1b have not been linked to transient integrin activation. Insight into the functional reversibility of integrin activation pathways will help to better understand the effects of antiplatelet agents.

## 1. Molecular Concept of Integrin Activation

Integrin αIIbβ3, previously known as glycoprotein (GP)IIb/IIIa, is preferentially and highly expressed on resting platelets with 60,000–80,000 copies per cell, with additional copies from the open canicular system and granules appearing upon platelet activation [1,2,3]. The α and β integrin peptide chains typically consist of a large extracellular part, a transmembrane spanning region and a short intracellular tail. The αIIb extracellular part contains an N-terminal β-propeller domain, a thigh domain and two calf domains. The extracellular β3 part is composed of an A domain, a plexin/semaphorin/integrin domain, four epidermal growth factor (EGF) domains and a membrane-proximal β-tail domain. Together, the extracellular αIIb β-propeller and β3 A domains form the integrin head [2,4].

In the early 2000s, the crystal structure was resolved of αvβ3 as a typical integrin [5]. By using electron microscopy, three conformations of the extracellular domains of the structurally similar integrin αIIbβ3 were demonstrated with low, intermediate and high-affinity for its ligands [6,7]. The conformation changes appeared to be accompanied by exposure of activation epitopes, known as ligand-induced binding sites (LIBS) [4,8,9]. Structural analyses suggested that, in the resting state, the membrane-proximal regions of the cytoplasmic α and β tails along with the helixes in the transmembrane regions form a complex, which locks or clasps both integrin chains [6,7]. Agonist-induced integrin activation (described as inside-out signaling) leads to unclasping in an equilibrium-controlled process, suggesting reversibility. In-depth descriptions of these structural changes of integrins are provided elsewhere in excellent reviews [4,10].

Similarly to the integrins of other cell types, the intracellular tails of αIIbβ3 form part of an adhesion complex linked to the actin cytoskeleton, which includes isoforms of kindlin and talin, several small molecule GTP-binding (SMG) proteins and a number of protein kinases [11,12].

The integrin heterodimer with αIIb and β3 subunits resembles other integrins in that the ‘unclasping’ conformational change is needed for increased ligand binding affinity. It has become clear that in the bent-closed (clasped) and the extended-closed conformations, association of the transmembrane regions of αIIb and β3 hides the extracellular ligand-binding site. In the extended open conformation, when the α and β chains unclasp, the ligand-binding MIDAS site (metal ion-dependent adhesion site) becomes exposed [2,3]. Thus, in its activated form αIIbβ3 serves to bind ligands as fibrinogen, fibrin and von Willebrand factor (VWF). In the cytosol, the integrin association with talin-1 and kindlin-2/3 was found to be indispensable for the activated conformational change and the ligand binding [12,13]. Other, less abundant platelet integrins such as α2β1 (collagen receptor) and α6β1 (laminin receptor) may undergo similar conformational changes as a requirement for ligand binding [14,15].

Recently, a general model of mechanical force-dependent integrin activation has been proposed, in which the actomyosin cytoskeleton mechanically pulls and transduces a force via talin-1, and possibly kindlin, to open the resting (bent) integrin conformation, which thereby allows an integrin to bind its ligands [16]. In this model, the bent-closed state is thermodynamically favored, while cytosolic integrin inactivators such as moesin, filamin A and sharpin (all highly expressed in platelets [17]) can destabilize the active integrin structure with or without mechanical actomyosin forces [16]. A stable ligand binding to the activated integrin conformation is thought to be achieved by avidity-based clustering of multiple integrins [18].

An important implication of this model is that it considers the mechanism of integrin activation as an intrinsically reversible process. In contrast, earlier literature supposed that integrin αIIbβ3 activation in response to agonists is an irreversible event, leading to permanent platelet aggregation and adhesion. Yet, over the years, an increasing number of reports has shown reversibility of the platelet aggregation process. In the present paper, we use the terms ‘reversible integrin activation’ and ‘integrin in-activation’ from a functional perspective. Thus, integrin in-activation stands for the secondary inability of αIIbβ3 to bind fibrinogen or antibodies directed at its activated conformation, such as observed in connection to platelet disaggregation. Of note, to which extent the secondary absence of ligand binding is caused by structural reversal of the integrin chains to the bent-closed conformation is unclear.

Indirect support for reversibility of integrin activation comes from in vivo studies by the Philadelphia group, showing that in a microvascular thrombus loosely adhered platelets in the outer shell frequently detach from the thrombus core of densely packed platelets [19]. In the following sections, we discuss the agonists and signaling pathways that result in such reversibility. We explore the conditions that lead to platelet disaggregation, platelet detachment from a thrombus, and thrombus instability. In addition, we mention the relevance of this process for cardiovascular health and disease.

## 2. Reversible Integrin αIIbβ3 Activation and Inside-Out Signaling

For long, integrin αIIbβ3 activation has been considered a hallmark of platelet responsiveness. The activated integrins on adjacent platelets bind with high affinity to the bivalent fibrinogen molecules, which results in the formation of platelet aggregates held together by αIIbβ3-fibrinogen bridges [2]. Under high-shear flow conditions, also the integrin-dependent interaction with VWF can contribute to the aggregate formation [20]. Low-level signaling through the GPIb-V-IX complex can support the binding of fibrinogen to αIIbβ3 and hence platelet aggregation [21].

**Figure 1:**
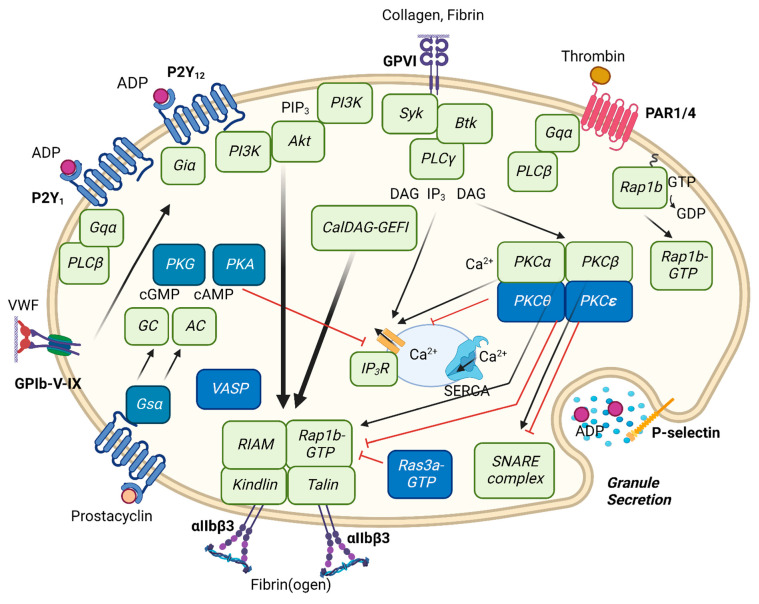
Key signaling pathways in platelets linked to (reversible) integrin activation αIIbβ3 and platelet aggregation. For explanations, see text. In short, the IP receptor for prostacyclin inhibits platelets via adenylyl cyclase (AC), while gaseous nitric oxide inhibits platelets via guanylate cyclase (GC); the formed cAMP and cGMP activate protein kinase A (PKA) and protein kinase G (PKG), respectively. As platelet adhesion receptors, integrin and GPIb-V-IX interact with fibrinogen, fibrin and von Willebrand factor (VWF). The purinoceptors P2Y_12_ and P2Y_1_ operate after autocrine release of ADP; P2Y_12_ acts via the G protein Giα, inhibiting AC but stimulating phosphoinositide 3-kinase (PI3K). On the other hand, P2Y_1_ signals via Gqα which stimulates phospholipase C (PLC), causing Ca^2+^ release and activation of protein kinase C (PKC). As a strong platelet agonist, thrombin also activates Gqα-coupled receptors, namely PAR1 and PAR4. The collagen receptor GPVI activates a protein tyrosine kinase pathway involving Syk and Btk, leading to downstream activation of PLC and PI3K isoforms, the latter stimulating Akt protein kinase. The signaling toward activation of αIIbβ3 furthermore involves the small GTPase-regulating proteins CalDAG-GEFI (calcium and diacylglycerol regulated guanine nucleotide exchange factor I), Ras3a and Rap1b. The cytoskeleton-linked signaling is completed by kindlin and talin isoforms. Black arrows show relative strength of pathways to integrin activation; red arrows connected to blue-boxed proteins represent inhibitory pathways.

The vast majority of signaling receptor agonists is capable to induce platelet aggregate formation [22,23]. These include agonists of G-protein coupled receptors (GPCRs), linked to the signal-transmitting Gqα and Giα proteins, and also agonists of immunoreceptor tyrosine-based activation motif (ITAM)-linked receptors (ILRs), such as the collagen receptor glycoprotein VI (GPVI). Accordingly, to mention the best-known ones, human platelet stimulation with epinephrine (via α2A receptors), ADP (via P2Y_1_ and P2Y_12_ receptors), thromboxane A_2_ (TXA_2_, via TP receptors), collagen (via GPVI and α2β1) and thrombin (via protease-activated receptors PAR1 and PAR4) all induce integrin αIIbβ3 activation and aggregate formation (Figure 1).

Whereas the agonist-induced signaling pathways to αIIbβ3 activation (inside-out signaling) are well understood, the subsequent events leading to (ligand-induced) integrin clustering are less clear [13]. Depending on such clustering, patches of ligand-occupied αIIbβ3 integrins can also evoke signaling responses. This is known as integrin outside-in signaling, a process that involves several protein tyrosine kinases as well as signaling adaptors and cytoskeletal components [18]. By convention, outside-in signaling is required for the spreading of platelets on a fibrinogen surface and for the contraction of a fibrin clot. It is likely, but not definitively proven, that outside-in signaling contributes to the stabilization of platelet aggregates and formed thrombi [24,25].

Below we provide a comprehensive overview on the signaling actions triggered via GPCRs or ILRs that link to reversible or transient activation of integrin αIIbβ3 and to platelet disaggregation. Herein, we focus on specific receptors, downstream signaling components, protein phosphorylations and the release of secondary mediators.

## 3. ADP Receptor Stimulation

The two platelet receptors for ADP, i.e., P2Y_1_ (gene *P2RY1*) linked to Gqα, and P2Y_12_ (*P2RY12*) linked to Giα [26], are both required for the full induction of platelet aggregation, such as monitored by light transmission aggregometry [27,28]. Upon ADP stimulation, P2Y_1_ induces a signaling route to phospholipase Cβ (PLCβ) and protein kinase C (PKC); whereas P2Y_12_ causes inhibition of adenylate cyclase and activation of phosphoinositide 3-kinase (PI3K) isoforms (Figure 1) [29].

Several reports indicate that the platelet aggregation induced by ADP (as a ‘weak’ agonist) is particularly sensitive to disaggregation (Table 1). Several drugs have been described that secondarily reverse the aggregation with ADP, in particular the αIIbβ3 antagonists (abciximab, lamifiban, SR121566, tirofiban) [30,31]. These drug effects suppose that the agonist-induced binding of fibrinogen to αIIbβ3 is reversible, in a way that integrin antagonists can compete with the ligand. Under both static and flow conditions, it has indeed been shown that integrin inhibitors compete with fibrinogen and thereby reverse platelet aggregation [32,33].

Other well-studied aggregation-reversing agents are blockers of the P2Y_1_ or P2Y_12_ receptors and the enzyme apyrase, which degrades ADP. Flow cytometric evidence has shown that, following ADP-induced αIIbβ3 activation (measured as FITC-PAC1 mAb binding to platelets), the subsequent blockage of P2Y_1_ or P2Y_12_ receptors or later ADP removal resulted in a lower extent of αIIbβ3 ligand binding [31,34]. Similarly, in platelets from patients with a defect in P2Y_12_ receptors, often a reversible ADP-induced aggregation is observed, even at high ADP concentrations above 10 μM [28]. The same holds for patients who are treated with P2Y_12_ receptor blockers. Accordingly, platelet activation via both ADP receptors appears to be required for a persistent aggregation response, such as has been concluded earlier [35].

Another way to downregulate ADP-induced platelet responses is via ecto-nucleotidases such as CD39, which hydrolyzes ATP and ADP into AMP and adenosine [36]. In an elegant approach to make use of ecto-nucleotidases, recombinant CD39 was fused with a single chain antibody fragment recognizing the activated αIIbβ3, named Targ-CD39 [37]. This allowed the CD39 to only hydrolyze the ADP that is released from activated and aggregated platelets in a thrombus. In a mouse model of cardiac ischemia/reperfusion, the platelet-binding Targ-CD39 construct caused protection of the reperfused tissue [38].

Other drugs that secondarily reverse the ADP-induced platelet aggregation and αIIbβ3 activation appeared to be blocking agents of PI3Kβ [31], which confirms the key role of the PI3K pathway downstream of P2Y_12_. Furthermore, secondary platelet inhibition with iloprost or the replacement of Mg^2+^ by Ca^2+^ (affecting the integrin MIDAS domains) were found to reverse the ADP-induced platelet aggregation (Table 1). The observations that P2Y_1_ and P2Y_12_ blocking as well as PI3K inhibition leads to disaggregation implies that both the Gqα and Giα signaling pathways are required for a persistent functional integrin activation and ligand binding. This might imply a transiency of the actomyosin force-dependent conformation change of integrins, although this has not yet been proven.

## 4. Collagen GPVI Receptor Stimulation

Platelet stimulation with collagen or collagen-related peptides induces a signaling pathway via GPVI and the ITAM-linked FcR g-chain, involving protein tyrosine kinases like Src, Syk and Btk [23,29]. As a result, PLCγ and PKC isoforms become activated as well as PI3K isoforms [39,40]. A noticeable aspect of the platelet aggregation with lower doses of collagen is that it relies on release of the autocrine mediators, ADP and TXA_2_ [22]. This can explain why the secondary inhibition of either αIIbβ3, P2Y_12_ or PI3K can cause reversion of a collagen-induced platelet aggregation response (Table 1). In agreement with this, in microfluidics tests where whole blood is flowed over collagen, the inhibition of autocrine mediators appeared to suppress the thrombus formation and to cause disaggregation of platelets from the formed thrombi [41].

A comparison of the roles of human and mouse GPVI in platelet aggregation and thrombus stability indicated that especially the blockage of human platelet GPVI led to disaggregation [42]. In mice, most markedly a deficiency in integrin β3 led to a transient collagen-mediated platelet aggregation and an unstable thrombus formation [43].

## 5. Thrombin PAR1 and PAR4 Receptor Stimulation

Thrombin activates human platelets via cleavage of the GPCRs, PAR1 and PAR4, both of which receptors are coupled to Gqα and accordingly induce a common signaling route to PLCβ and PKC stimulation (Figure 1) [26,29]. Both receptors are cleaved at the N-terminus to uncover a so-called tethered ligand. The ligand peptide sequence of PAR1 consists of the sequence of SFLLRN, which as a hexapeptide (thrombin receptor-activating peptide: TRAP6) can also activate the receptor; for PAR4 the corresponding sequence consists of AYPGKF. Human platelet activation by TRAP6 via the PAR1 receptor results in granule release and in αIIbβ3 activation, of which the latter process has been shown to be reversible (Table 1). On the other hand, this reversibility has not been reported for the PAR4 peptide AYPGKF or for thrombin.

In hemostasis and thrombosis, the generation of thrombin is in part triggered by vascular-exposed tissue factor. Kinetic studies with flowed blood have shown that the role of tissue factor in platelet aggregation and thrombus formation is only short-term [44]. Interestingly, one report states that this role of tissue factor can depend on factor VII-activating protein (FSAP, gene *HAPP2*). Deletion of the *Happ2* gene in mouse appeared to reduce the thrombus-forming process, but did not cause thrombus instability [45]. As described below, the initial role of tissue factor in thrombin generation can be taken over by procoagulant platelets, exposing phosphatidylserine [22].

## 6. Integrin αIIbβ3 Regulation by Other Extracellular Proteases

A variety of proteases present in the blood plasma and in the platelet cytosol are involved in the sustained integrin αIIbβ3 activation and platelet aggregation. The majority of proteases must first be activated for instance by proteolysis, as in the case of thrombin (generated from prothrombin) and plasmin (from plasminogen) [22,46].

Regarding persistent αIIbβ3 activation, a still incompletely understood role is played by the family of zinc-dependent matrix metalloproteinases (MMP) [47]. The isoforms MMP1, 2, 9, 12, 13 and 14 are all known to modulate the platelet activation processes [46]. Both MMP1 and MMP9 enhance platelet aggregation induced by collagen under flow [48,49]. The mechanism may rely on a proteolytic cleavage of PAR1 or other receptors [46]. Additionally, the isoform MMP2 primes for platelet activation [50], which is also the case for MMP12 [51]. The membrane-bound isoform MMP14 may induce platelet responses by a cleavage of pro-MMP2 and pro-MMP13 [52].

## 7. Integrin αIIbβ3 Regulation via Protease-Dependent Receptor Cleavage

Receptor cleavage is another way to regulate integrin activation. An example is provided by the platelet-expressed proteases ADAM10 and ADAM17 (for: a disintegrin and metalloprotease), which function as sheddases for the extracellular domains of GPVI (ADAM10) and GPIbα (ADAM17) [53,54]. It has appeared that the ADAM-induced receptor cleavages are prominent in highly activated platelets, which can provide another mechanism to abrogate the aggregation response [55].

In highly activated platelets, i.e., by thrombin plus collagen stimulation, prolonged and high cytosolic Ca^2+^ rises lead to opening of anoctamin-6, which is a phospholipid and ion channel, and thereby to the surface exposure of procoagulant phosphatidylserine, which promotes the assembly of coagulation factor complexes [22]. Accompanying the procoagulant response is the Ca^2+^-dependent prolonged activation of calpains, leading to cleavage of the intracellular domain of integrin β3 [56], as well as of several proteins that are required for integrin activation (Src, filamin-A, talin-1, kindlin-3) [57]. Accordingly, in the highly activated platelets, αIIbβ3 becomes inactivated (abolishment of PAC1 mAb binding) and the aggregation response is blocked [56,58]. Uncontrolled calpain activation thus provides another pathway for functionally switching off αIIbβ3 integrins [34].

## 8. Reversible Integrin Activation and Thrombus Instability

An accepted model of murine (microvascular) thrombus formation describes the thrombus architecture as composed of an inner core with highly activated and contracted platelets, which is surrounded by a shell region with low-activation, loosely and transiently adhered platelets [59]. This heterogeneity has been explained by a different exposure of platelets to agonists like collagen, thrombin, ADP and TXA_2_ together with differences in shear forces. In the core region, tissue-factor induced thrombin generation contributes to a PAR- and fibrin-dependent platelet contraction. On the other hand, the second mediators ADP and TXA_2_ will act as main platelet agonists in the shell region, in which the outflow of mediators restricts the agonist concentrations [59,60].

Another form of heterogeneity has been observed in thrombi generated on collagen under flow conditions. Here, patches of aggregated platelets are formed, staining for fibrinogen, and separated from these single, balloon-shaped platelets with phosphatidylserine exposure and not binding fibrinogen [61]. It has been argued that the integrin inactivation of those platelets helps to stimulate the coagulation process [46].

Whole-blood flow chamber experiments have further shown that the platelets which disaggregate from a preformed thrombus lose their ability to bind fibrinogen and hence inactivate their integrins [31]. In terms of thrombus formation, the reversibility of (ADP-induced) platelet integrin activation likely contributes to events as thrombus instability and dissolution. However, it needs to be stated that, in vivo, also other processes will be involved in thrombus instability, such as local high shear forces, fibrinolysis and other proteolytic activities in an occluding artery. To which extent reversible integrin activation is important in arterial thrombosis still needs to be determined.

In mice, a deficiency of either P2Y_1_ or P2Y_12_ was found to affect arterial thrombus formation in vivo, and also caused instability of thrombi that still formed (Table 2) [62,63,64]. The same applied to the infusion of P2Y_12_ antagonist, ticagrelor [65]. That P2Y_12_ receptors have a thrombus-stabilizing role was also concluded from in vivo studies with *Apoe*^-/-^ mice, where plaque-induced thrombus formation and stability were impaired upon receptor blockage [66]. Additionally, in mouse models, application of a reversible P2Y_12_ antagonist was found to dissolve the preformed platelet thrombi [67]. Together these findings point to major roles of the two platelet ADP receptors in stable arterial thrombus formation. Although the extent of activation of αIIbβ3 cannot be followed in the in vivo conditions, functional reversibility of the integrin activation is a reasonable explanation of the results.

## 9. Integrin αIIbβ3 Regulation by Intracellular Signaling Molecules

Several signaling pathways are at the center of platelet integrin activation regulation, and for some of these there is evidence for reversibility.

### 9.1. PLC and PKC Isoforms

Stimulation of GPCR- (via Gqα) and ILR-dependent (via Syk) signaling routes leads to activation of isoforms of PLCβ/γ and PKC, which are essential components in platelet responses like granule secretion, integrin activation and platelet aggregation [22]. The isoforms of PKC are broad-spectrum protein kinases, of which in particular PKCα, PKCε and PKCθ have been studied in platelets [68,69]. The platelets from PKCα-deficient mice are strongly impaired in aggregation and thrombus formation [70], which leads to the conclusion that PKCα is an essential protein kinase for achieving integrin αIIbβ3 activation, such as for example induced by phorbol esters. On the other hand, in mice lacking PKCε or PKCθ, platelet aggregation and thrombus formation were increased under certain conditions (Table 2) [71,72,73].

### 9.2. PI3K Isoforms

Enzymes of the PI3K family phosphorylate phosphoinositide lipids at the 3′ position of the inositol ring, in particular to produce phosphatidylinositol 1,4,5-trisphosphate (PIP_3_). Well studied in relation to platelet integrin activation are the class-I isoforms PI3K α, β and δ [74]. Upon PI3K activity, the produced PIP_3_ attracts key signaling proteins with so-called pleckstrin homology (PH) domains to the membrane. Earlier studies have indicated that the activity of PI3K isoforms is required for a perpetuated integrin activation [75,76]. Pharmacological analysis indicated non-redundant roles of PI3Kα and PI3Kβ in the GPVI-induced platelet activation and thrombus formation, in particular by contributing to Rap1b activation [40]. Furthermore, a post-treatment of collagen- or ADP-induced platelet aggregates with the PI3Kβ inhibitor TGX-221 appeared to result in immediate disaggregation and reversal of the binding of fibrinogen or PAC1 mAb to integrin αIIbβ3 (Table 1). Additionally, murine deficiency in either PI3Kα or PI3Kβ led to smaller sized arterial thrombi and to reversible platelet aggregation responses (Table 2).

### 9.3. Akt Isoforms

Protein kinases of the Akt family (alternatively named protein kinase B) provide major PIP_3_-binding proteins in the PI3K signaling cascade (Figure 1). From both in vivo and in vitro studies, it appeared that in mouse platelets the three isoforms Akt1, Akt2 and Akt3 contribute all to aggregate formation and thrombus stability (Table 2) [77,78,79]. In particular the deficiency of Akt1 resulted in an impaired collagen-induced platelet aggregation [77,80,81]. On the hand, murine deficiency in either Akt2 or Akt3 led to a disaggregation of platelets after stimulation with (low doses of) ADP- or thrombin-receptor agonists [78,79]. Summarizing this places the PI3K-Akt pathway as an controlling route for (persistent) platelet aggregation.

### 9.4. Small GTPases and Integrin Regulation

Platelets contain almost 500 small GTP-binding proteins and regulators [17]. These include effector GTP-binding proteins, activating guanine nucleotide exchange factors (GEF), and signal-abrogating GTPase-activating proteins (GAP). Several of these proteins are considered to be crucial for integrin αIIbβ3 activation and can be linked to functional integrin reversibility. Relevant are: (a) Rap1b; (b) its activator CalDAG-GEFI (calcium and diacylglycerol regulated guanine nucleotide exchange factor I; gene *RASGRP2*); (c) Rasa3 as a Rap1b-inactivating GAP; (d) the protein ARHGEF10; (e) the small GTPase RhoA; and (f) TC21/RRas (*RRAS2* gene).

**Rap1b** undergoes a GDP for GTP switch in response to essentially all platelet agonists, resulting in its active, GTP-bound state [82,83]. The GTP-bound Rap1b is known to support αIIbβ3 activation through RIAM (Rap1-interacting adaptor molecule), which facilitates the integrin interaction with talin and kindlin on the plasma membrane [12,84]. Depending on the type of platelet trigger, Rap1b can be activated via two signaling pathways, one via a Ca^2+^-dependent CalDAG-GEFI activation route, and also via another slower but sustained PKC route [85,86,87]. The second route may require ADP co-stimulation via P2Y_12_ and PI3K [88,89]. In mouse, Rap1b deficiency caused strong defects in integrin inside-out and outside-in signaling [90,91], as well as in TXA_2_ release and granule secretion [91,92]. So far, there is no evidence for a particular role of Rap1b in aggregate stabilization, although its role in arterial thrombosis is clear [90].

**CalDAG-GEFI**, as a main Rap1b activator, becomes active via agonist-induced rises in cytosolic Ca^2+^. The protein has a low-affinity binding site for diacylglycerol, which makes a regulation via physiological levels of diacylglycerol unlikely [93]. CalDAG-GEFI has been identified as a rapid and reversible control switch for integrin αIIbβ3 activation. In human, a loss-of-function mutation resulted in aberrant platelet aggregation that was associated with bleeding. Supporting evidence for such a role of CalDAG-GEFI comes from *Rasgrp2* knockout mice. Platelets from these mice were severely hampered in their ability to aggregate with multiple agonists, and to contribute to arterial thrombus formation [85,94]. No thrombus instability has been reported, such in contrast to P2Y_12_ inhibition (Table 2). An alternative, CalDAG-GEFI-independent route to integrin activation is provided by the slower diacylglycerol and PKC-dependent route [86].

**Ras3a** has been identified in platelets as key deactivator of Rap1b, catalyzing the hydrolysis of Rap1b-GTP to GDP [95]. Platelets from mice with a mutant Rasa3 form appeared to be hyperactive, suggesting that this protein keeps the circulating platelets in a quiescent state by restraining the CalDAG-GEFI and Rap1b signals [95]. It is suggested that P2Y_12_ signaling (via PI3K) results in Rasa3 inhibition, which further enables Rap1b-dependent platelet aggregation and thrombus formation. Autocrine released ADP indeed is a potent enforcer of platelet aggregation via P2Y_12_ receptors [29].

In mice lacking **ARHGEF10**, platelet stimulation via ILRs or GPCRs resulted in aggregation responses which gradually declined. In vivo experiments pointed to an unstable arterial thrombus development and a longer tail bleeding time [96]. Mechanistically, ARHGEF10 is considered to regulate the activation of RhoA.

**RhoA** is known to have a role in αIIbβ3-induced outside-in signaling, and hence supports platelet spreading, cytoskeletal reorganization and clot retraction [97]. In mouse, megakaryocyte/platelet-specific RhoA deficiency thus led to impaired platelet activation responses [98].

**TC21/RRas** is required for full GPVI-induced platelet responses, up to now according to one paper. The reported impairments include tyrosine phosphorylation, integrin activation and secretion, as well as thrombus instability in vivo, as established in deficient mice [99]. Evidence is also provided that this small GTP-binding protein can control the activation of Rap1b.

## 10. Platelet Inhibition by Protein Kinases A and G

Two endothelial-derived mediators, i.e., prostacyclin and nitric oxide, antagonize most platelet responses, including integrin αIIbβ3 activation and aggregate formation [29]. Prostacyclin acts via binding to a GPCR linked to Gsα, which stimulates adenylate cyclase to produce cAMP. This second messenger triggers the broad spectrum Ser/Thr protein kinase A (PKA) [100]. Nitric oxide diffuses across the platelet membrane, and directly stimulates guanylate cyclase to form cGMP, which activates protein kinase G (PKG) (Figure 1). Via stimulation of PKA and PKG a large number of proteins becomes phosphorylated, which thereby ensures a multi-targeted way of platelet inhibition, including proteins linked to integrin activation [101].

A particular phosphorylation substrate of both PKA and PKG, related to platelet inhibition, is vasodilator-stimulated phosphoprotein (VASP), which regulates the actin cytoskeletal dynamics [102]. In VASP-null platelets, it was observed that the cAMP- and cGMP-dependent inhibition of platelet aggregation was abolished, but not the secretion response [103]. In wild-type mice, VASP can form a complex that regulates Rap1b inhibition [104]. Of clinical interest, VASP phosphorylation at Ser^239^ is a standard method to establish PKA- and P2Y_12_-dependent phosphorylation events [105]. Both prostacyclin and nitric oxide can suppress the agonist-induced activation of Rap1b [82,106].

The two platelet-inhibitory PKA and PKG pathways are halted by a negative feedback loop of cAMP and cGMP hydrolysis through cyclic nucleotide phosphodiesterases (PDE). Of these, PDE2 and PDE3 mainly lower cAMP levels, while PDE5 lowers cGMP [107]. The feedback pathway plays a role upon platelet stimulation through the Giα-coupled receptor P2Y_12_, which leads to inhibition of adenylate cyclase and cAMP can no longer rise [108]. Additionally, the activity of PDE3 is increased upon thrombin stimulation [109].

The importance of PKA in suppressing platelet aggregation activation becomes clear from the fact that the secondary application of iloprost (a prostacyclin analogue) can reverse the integrin activation in response to multiple agonists (Table 1) [110]. In addition, gain-of-function mutations in the Gsα protein lead to elevated platelet cAMP levels, lower aggregate formation and a bleeding phenotype [111], whereas loss-of-function mutations leads to an impaired platelet inhibition with iloprost [100].

## 11. Concluding Remarks and Relevance

Overviewing the molecular signaling events that link to a reversible integrin αIIbβ3 activation, these are especially related to the ADP receptor pathways, including the conditions in which ADP acts as an autocrine mediator. The signaling alone via P2Y_1_ or P2Y_12_ receptors shows a certain transiency, leading to a transient way of integrin binding to its ligands. One can tentatively conclude that, to assure permanent integrin activation, the continued presence of ADP is essential acting via both P2Y receptors. Downstream of these receptors, especially the signaling via PI3K and Akt isoforms ensures irreversibility of the platelet aggregation process. In addition, the reversibility of collagen-induced (via GPVI) and TRAP6-induced (via PAR1) integrin activation can be linked to a transient PI3K activity and/or transient P2Y receptor functions. So far, there is no evidence for reversibility due to low PKC, CalDAG-GEFI or Rap1b activities, thus suggesting that the switch for a reversible offset of integrin αIIbβ3 ligand binding resides early in the signaling cascade.

From a (patho)physiological perspective, thrombus consolidation is a final stage of hemostatic plug formation. Platelet exposure to ‘strong’ agonists, like collagen and thrombin, appears to be required for such consolidation. The ‘weaker’ agonist ADP appears to extend and also restrict the initiating roles of collagen and thrombin, e.g., by forming the ‘loose’ outer shell of an intravascular thrombus. The fact that at least part of these ADP effects—in terms of integrin activation and platelet aggregation—are reversible may explain the success of anti-P2Y_12_ drugs in thrombus suppression and possibly reversion. At the same time, realizing this, it is not a surprise that clinically used P2Y_12_ antagonists have bleeding as a side effect. Improved insight into the transiency of integrin-dependent molecular pathways may thus help to better understand the (patho)physiology of hemostasis and thrombosis.

In this respect, the high αIIbβ3 expression and platelet activation recently observed in diabetic patients [112,113] may point to a shifted equilibrium in the ability to integrin ligand binding. It has been demonstrated that in diabetic platelets the force-induced integrin αIIbβ3 activation increases in a PI3K-dependent way, which resulted in an exaggerated shear-dependent platelet adhesion [114].

**Table 1:** Drugs/interventions reported to reverse human platelet aggregation in response to given agonists.

Agonist	Reversing Inhibitor	Reversing Pathway	Reference
ADP	tirofiban, abciximab	αIIbβ3 antagonism	[30]
ADP	SR121566	αIIbβ3 antagonism	[115]
ADP	Gas6 depletion	TAM antagonism	[116]
ADP	citrated PRP plus CaCl_2_	Ca^2+^/Mg^2+^ replacement	[117]
ADP, shear	lamifiban	αIIbβ3 antagonism	[118]
ADP, collagen	ticagrelor	P2Y_12_ antagonism	[31,119]
ADP, collagen	TGX-221, wortmannin	PI3K antagonism	[31]
ADP, collagen	iloprost	cAMP elevation	[31]
ADP, TRAP6	αCD62P antibody	P-selectin blockage	[120]
TRAP6	iloprost (+tirofiban)	cAMP elevation	[110]
PAR1p	wortmannin	PI3K antagonism	[121]
PAR4p	2-MeSADP	P2Y antagonism	[86]

Abbreviations: TAM, Tyro, Axl and Mer receptors; PAR1p, PAR1 activating peptide.

**Table 2:** Selection of genetic defects in mouse resulting altered arterial thrombus formation whether or not accompanied by platelet disaggregation or embolization in vivo or in vitro.

Gene Defect	Protein Defect	Thrombus Formation	Disaggregation or Embolization	References
*Akt1*	protein kinase Akt1	↓	no	[77,80,81,122]
*Akt2*	protein kinase Akt2	↓	**yes**	[78]
*Akt3*	protein kinase Akt3	↓	**yes**	[79]
*Arhgef10*	GEF Rho-GEF10	↓↓	**yes**	[96]
*Cd18*	integrin β2 (CD18)	↓	no	[123]
*Gp6*	GPVI receptor	↓	no (human **yes**)	[42]
*Happ2*	factor VII activating (FSAP)	↓	**yes**	[45]
*Itga2*	integrin α2	0 or ↓	no	[124,125,126]
*Itga2b*	integrin αIIb	↓↓	no	[127]
Itga6	integrin α6	↓↓	no	[128]
*Itgb1*	integrin β1	↓	**yes**	[129,130,131]
*Itgb3*	integrin β3	↓↓	**yes**	[43]
*P2ry1*	P2Y1 receptor	↓↓	**yes**	[62,63]
*P2ry12*	P2Y12 receptor	↓↓	**yes**	[62,64,66,67,132]
*Pik3ca*	PI3K alpha	↓	no	[133]
*Pik3cb*	PI3K beta	↓↓	**yes** (U46619)	[134]
*Pik3cg*	PI3K gamma	↓↓	**yes** (ADP)	[31,135]
*Prkca*	PKC alpha	↓↓	no	[70]
*Prkcd*	PKC delta	0	no	[73,136]
*Prkce*	PKC epsilon	↑	no	[71]
*Prkcq*	PKC theta	↓ or ↑	no	[72,73,137,138]
*Rasa3*	GAP Rasa3	↑	no	[95]
*Rasgrp2*	GEF CalDAG-GEFI	↓↓	no	[132,139]
*Rap1b*	GTPase Rap1b	↓↓	no	[90]
*Rhoa*	GTPase RhoA	↓↓	no	[98]
*Rras2*	TC21/RRas	↓	**yes**	[99]
*Tln1*	talin 1	↓↓	no	[11,84]
*Treml1*	TLT-1	↓	no	[140]
*Vasp*	VASP protein	0	no	[103]

Gene defects leading to disaggregation or embolization are indicated in bold. Abbreviations: GAP, GTPase activating protein; GEF, GTP exchange factor. See also text. Consequence of gene defect on thrombus formation is indicated as decrease (↓), strong decrease (↓↓), no effect (0) or increase (↑).

## Data Availability

This review does not contain novel, unpublished data.
